# Indications, Functional Outcomes, Return to Sport and Complications of Anterior and Lateral Approaches for Total Ankle Arthroplasty: A Comprehensive Review

**DOI:** 10.3390/healthcare13070841

**Published:** 2025-04-07

**Authors:** Michele Mercurio, Erminia Cofano, John G. Kennedy, James J. Butler, Antonio Zanini, Olimpio Galasso, Giorgio Gasparini, Alberto Marangon

**Affiliations:** 1 Department of Orthopaedic and Trauma Surgery, Magna Graecia University, 88100 Catanzaro, Italy; michele.mercurio@unicz.it (M.M.); gasparini@unicz.it (G.G.); 2 Research Center on Musculoskeletal Health, MusculoSkeletal Health@UMG, Magna Graecia University, 88100 Catanzaro, Italy; 3 Division of Foot and Ankle Surgery, NYU Langone Health, New York, NY 10002, USA; john.kennedy@nyulangone.org (J.G.K.); james.butler@nyulangone.org (J.J.B.); 4 S. Anna Clinica Institute, 25127 Brescia, Italy; antoniozanini.mn@libero.it; 5 Department of Medicine, Surgery and Dentistry, University of Salerno, 84081 Baronissi, Italy; ogalasso@unisa.it; 6 Clinic San Francesco, 37127 Verona, Italy; alberto.marangon@me.com

**Keywords:** total ankle replacement, fixed bearing, mobile bearing, AOFAS, malleolar fracture, return to activity

## Abstract

Ankle osteoarthritis (OA) is a degenerative condition that impacts quality of life. Total ankle replacement (TAR) represents a significant advancement in orthopedic surgery. **Objectives**: The purpose was to provide an overview of the indications, outcomes, and complications of anterior and lateral surgical approaches in TAR, as well as return to sport following surgery. **Methods**: The PubMed, MEDLINE, Scopus, and Cochrane Central databases were searched. The keywords used were “total ankle arthroplasty”, “total ankle replacement”, “ankle anterior approach”, “ankle lateral approach”, “outcomes”, “return to sport”, and “complications”, and the search included articles published from 2014 to 2024. **Results**: Successful functional outcomes, return to athletic activity, and return to the previous level of sports performance after surgery have been reported at rates of over 60%. The anterior approach restores the normal tibial slope but presents a high risk of wound-healing complications and medial malleolar fractures. The lateral approach allows an anatomic placement of the implant, but it is associated with fibular complications and a high risk of revision surgery. Return to sport is feasible in low-impact sports such as cycling, swimming, and dancing. **Conclusions:** The anterior and lateral approaches for TAR yielded satisfactory functional outcomes and rates of return to athletic activity. Different intra- and post-operative complications and revision surgery should be managed properly to optimize outcomes.

## 1. Introduction

Ankle osteoarthritis (OA) is a debilitating, degenerative condition that significantly impacts quality of life and has an estimated incidence of 30 per 100,000 people annually [1]. Although OA in the hip or knee is usually primary OA, the most common etiology in the ankle is post-traumatic OA [2] due to osteochondral fractures or ligament instability. Other causes include rheumatoid arthritis, osteonecrosis, hemophilia, infections, and gout, and 30% of cases are idiopathic. These conditions result in pain, dysfunction, and impaired mobility.

The mental and physical disability associated with end-stage ankle OA is equivalent to that of hip and knee OA in terms of its impact on the patient’s quality of life [3,4]. Traditionally, ankle arthrodesis was the standard surgical procedure for treating serious conditions. However, it is not without complications; it may lead to non-union, malunion, increased oxygen consumption, and degeneration of adjacent joints, leading to future problematic arthritis both distally and proximally to the ankle [5]. Total ankle replacement (TAR) represents a significant advancement in orthopedic surgery, offering new treatment opportunities. TAR preserves the joint’s movement and function, and patients who have undergone TAR have better health-related quality of life than those who have undergone ankle arthrodesis, with an equal risk of complications and re-operations [6,7].

The history of TAR is marked by decades of innovation, trial, and error. The first functional TAR was performed by Lord and Marotte in 1973, utilizing a reversed hip-prosthesis concept [8]. This early design involved placing an inverted hip stem in the tibia and a cemented acetabular cup in the calcaneus, replacing the talus. In the late 1970s and 1980s, first-generation TAR designs began to emerge. These implants were typically two-component systems made of metal and polyethylene and were often fixed with polymethylmethacrylate (PMMA) cement [9]. While these systems showed some promise, they were plagued by issues such as aseptic loosening, osteolysis, and instability [10]. Lessons learned from these shortcomings led to the development of second-generation TAR systems in the 1990s and early 2000s. These systems emphasized cementless fixation, reduced bone resection, and improved biomaterials. These designs often included two-component fixed-bearing (FB) systems with polyethylene surfaces and porous coatings for bony ingrowth, addressing many of the previous generation’s limitations [11].

The advent of third- and fourth-generation TAR implants marked significant progress in the field. These generations introduced three-component mobile-bearing (MB) systems and semi-constrained FB systems designed to enhance joint stability and reduce wear. These modern implants were also associated with the adoption of more conservative surgical techniques that preserve critical bone structures and focus on retaining ligamentous integrity to maintain joint stability.

Overall, the results of TAR are satisfactory, and one of the most controversial aspects in terms of optimizing outcomes is the surgical approach. The anterior approach to TAR is the most widely used technique, offering excellent access to the ankle joint and the ability to visualize the tibial and talar cuts directly [5]. However, it carries potential risks, including risks of intraoperative medial malleolar fractures and potential injury to the neurovascular structures [12]. In contrast, the lateral approach, which requires a fibular osteotomy, provides enhanced lateral and posterior joint visualization and facilitates curved cuts that align with the ankle’s natural anatomy, potentially improving implant positioning and minimizing bone resection [13]. Moreover, unlike in shoulder, hip, or knee replacement, where different techniques can be applied using the same implant [14,15], the operative procedure for a TAR is intimately associated with the prosthetic design.

Also, post-operative management can be influenced by the surgical approach used, with different timings and protocols involved.

Attention to return to sport (RTS) has also increased in association with this type of prosthetic implant since sporting activity is practiced by increasing numbers of people. However, the ankle is significantly stressed during some sports, and this aspect influences the type of activity allowed after surgery.

The aim of revisiting the published literature is to provide an updated synthesis of new evidence for clinicians and healthcare decision-makers in an ever-evolving field.

The purpose of this review was to provide an overview of the indications, outcomes, and complications of anterior and lateral surgical approaches for TAR, as well as of return to sport following surgery.

## 2. Materials and Methods

**Figure 1:**
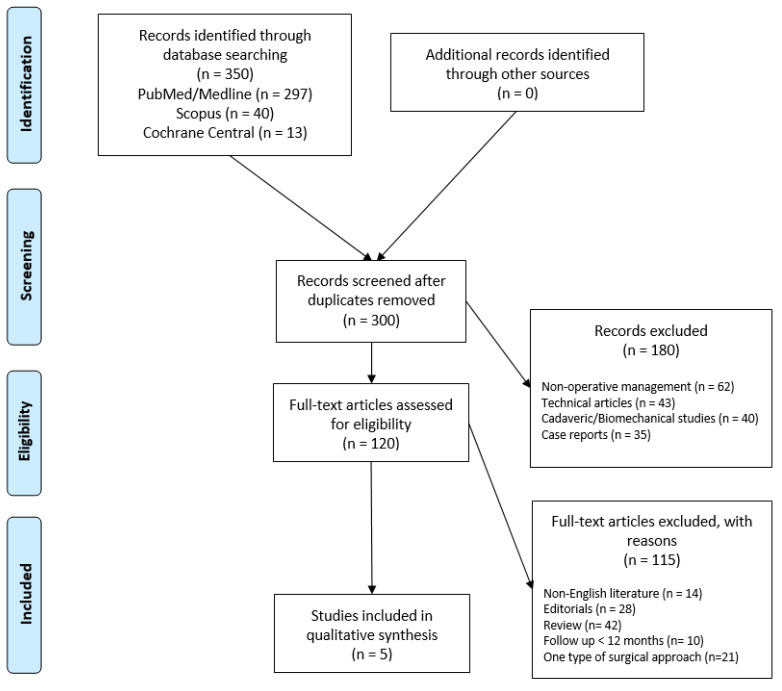
Preferred Reporting Items for Systematic Review and Meta-Analysis (PRISMA) flowchart for the search and the identification of included studies. Source: Moher et al. [15] For more information, visit https://www.prisma-statement.org/.

A comprehensive search was conducted in June and July 2024 using the databases PubMed, MEDLINE, Scopus, and Cochrane Central according to the Preferred Reporting Items for Systematic Reviews and Meta-Analyses (PRISMA) statement [15] (Figure 1).

The search terms “total ankle arthroplasty” OR “total ankle replacement” AND “ankle anterior approach”, AND “ankle lateral approach”, AND “outcomes” AND, “return to sport”, AND “complications”, were used in various combinations to identify relevant articles. Two different authors (MM and EC) screened the title and abstract to identify articles for inclusion, contacting a third senior author (GG) in cases of major discrepancies. The reference list including each article and the available gray literature at our institution were subsequently screened to identify any additional relevant articles.

In our study, we did a specific search of comparative studies between mobile and fixed bearing systems and between anterior and lateral surgical approaches, including both observational and experimental studies comprising case-control studies, cohort studies, and randomized controlled trials. The authors also considered reviews, case reports, technical notes, cadaveric or biomechanical studies, editorials, letters to the editor, and expert opinions for the discussion.

We included articles reporting the indications, outcomes, and/or complications of the most recently reported surgical techniques for TAR that had been published in the last 10 years and had a minimum mean follow-up of >12 months.

### 2.1. Inclusion and Exclusion Criteria

The inclusion criteria were applied during screening of the title, abstract, and full text according to the PICO, as follows: (1) population: patients who underwent surgery for ankle end-stage OA; (2) intervention: TAR (3) comparator: patients who underwent TAR with an anterior or lateral approach; (4) outcome: articles written in English reporting outcomes, complications, and return to sport with a minimum follow-up of 12 months. The exclusion criteria were as follows: (1) studies that considered only one type of surgical approach; (2) articles on revision of ankle replacement; (3) technical articles; (4) biomechanical studies.

### 2.2. Data Extraction and Quality Assessment

An assessment of methodological quality was conducted independently by two authors (MM and EC); cohort studies were assessed using the Modified Newcastle–Ottawa Quality Assessment Scale [16]. The discrepancies were resolved by consulting a senior reviewer (GG). Details of the quality assessment are shown in Table 1.

## 3. Biomechanics and Tribology of Total Ankle Replacement

During normal gait, the ankle joint absorbs significant forces, with the vertical load reaching up to 5.2 times the body weight [21]. In addition to vertical loading, the joint is subjected to anteroposterior and lateral shear forces, which are approximately two and three times body weight, respectively, generating varying patterns of stress across the articular surfaces. Ligamentous structures, such as the anterior talofibular ligament, the calcaneofibular ligament, and the tibiocalcaneal ligament, play a critical role in stabilizing the joint and limiting excessive movement during inversion and eversion [22]. The large surface area of the talus, approximately 11–13 cm^2^, helps efficiently distribute the forces across the joint, reducing localized stress and preventing rapid wear of the joint [23].

Changes in joint positioning, such as ankle dorsiflexion or plantarflexion, can significantly alter the contact patterns across the talar surface [24]. Ligamentous injuries can result in a decrease in talar contact area by 43% and an increase in peak pressures by 30%, underscoring the importance of intact ligamentous support in maintaining healthy joint biomechanics [25].

The TAR is designed to replicate the natural biomechanics of the ankle joint while accommodating its complex kinematics and load-bearing requirements. The biomechanics of TARs are influenced by several factors, including joint alignment, fixation, and articulation of the implant components. The tibial component interfaces with the polyethylene insert, which in turn articulates with the talar component. The tibial plafond, which is involved in the load transfer through the talus, must be adequately replicated to maintain proper joint motion and minimize wear of the implants. Additionally, the talar component must accommodate dynamic loading patterns that mimic the biomechanics of the natural talus to prevent abnormal stress concentrations that could lead to implant failure. Correct alignment is critical for minimizing wear patterns and ensuring that the implant functions in conjunction with the surrounding tissues and structures.

In a normal weight-bearing position, 77–90% of the load is transmitted through the dome of the talus via the tibial plafond, with the remaining load distributed through the medial and lateral talar facets [25]. Proper replication of this load distribution is essential to avoid the risk of stress concentrations that could result in implant loosening or subsidence. Malalignment of the implant components or improper positioning can alter the centroid of contact, leading to uneven load distribution and increased wear of the polyethylene insert, which may compromise the long-term function of the TAR [26].

Tribology, the study of friction, wear, and lubrication, plays a critical role in the design and performance of TARs [27]. Approximately 50% of TAR failures are linked to tribological issues, including wear, implant breakage, and loosening, making it a critical focus for improving implant longevity. Adhesion, abrasion, and fatigue are the primary wear mechanisms in TAR, with fatigue being influenced by implant geometry and patient biomechanics [27]. The frictional characteristics of TARs are determined by the materials used for the articulating components, as well as by the surface properties of those materials. A low coefficient of friction between the polyethylene insert and the metallic components is essential to minimize wear and reduce the risk of osteolysis. Materials such as cobalt−chromium alloys, titanium alloys, and ultra-high-molecular-weight polyethylene (UHMWPE) are commonly used in TAR components due to their high mechanical strength, wear resistance, and biocompatibility [28,29,30]. However, despite advances in materials, wear remains a challenge in TARs, with polyethylene particles continuing to contribute to wear-induced complications.

## 4. Indications and Contra-Indications for Total Ankle Replacement

The ideal patients for a TAR are patients at least 50 years of age with end-stage ankle OA, good bone stock, and no surrounding soft-tissue pathology. Body weight and body mass index (BMI) also play a role in suitability for TAR. Recent studies show no clear correlation between higher BMI and aseptic loosening, though increased BMI was associated with inferior musculoskeletal function and quality of life following TAR [31]. A BMI <30 kg/m^2^ is preferable, but patients with a significantly elevated BMI can be closely monitored and counseled regarding the risks. Patients with diabetes (HbA1c > 7) may face increased risks of infection and implant failure [32]. Alterations in the ipsilateral hindfoot, midfoot, and contralateral ankle are also degenerations that benefit more from TAR than from other solutions such as arthrodesis [31].

Active infection, Charcot neuro-arthropathy, osteonecrosis of the talar body or distal part of the tibia, severe peripheral vascular disease, an inadequate soft-tissue envelope, and substantial bone loss in the distal part of the tibia or the talus precluding adequate support for the arthroplasty components are absolute contraindications to TAR. On the other hand, peripheral neuropathy, severe lower-extremity malalignment, and marked osteoporosis are relative contraindications. Wound complications and poorer outcomes after TAR are associated with smoking habits [32].

### 4.1. Anterior Approach: Indications and Surgical Technique

There are specific indications for the anterior approach, such as being not obese/overweight, middle age or older age, and having reasonably mobile articulation, no significant comorbidities, low demands for physical activity, a well-aligned and stable hindfoot, no neurovascular impairment of the lower extremity, good bone stock, and good soft-tissue condition [33].

Usually, the surgery is performed in the operating room under spinal anesthesia, with the patient in the supine position, and with a pneumatic bandage at the thigh level in the absence of specific contraindications. The surgical approach has the following landmarks: medial and lateral malleoli and tibiotalar joint line.

An anterior longitudinal incision of 10 to 14 cm is made between the anterior tibial tendon and the extensor hallucis longus tendon to expose the retinaculum. The superficial peroneal nerve, which runs over the retinaculum, is identified. Between the anterior tibial tendon sheath and the extensor hallucis longus tendon sheath, the extensor retinaculum can be found lying down. The tibialis anterior vascular bundle, located behind the extensor hallucis longus or between the extensor hallucis longus and extensor digitorum longus, is carefully preserved. Once the ankle joint is exposed, capsulotomy and capsulectomy are performed.

**Figure 2:**
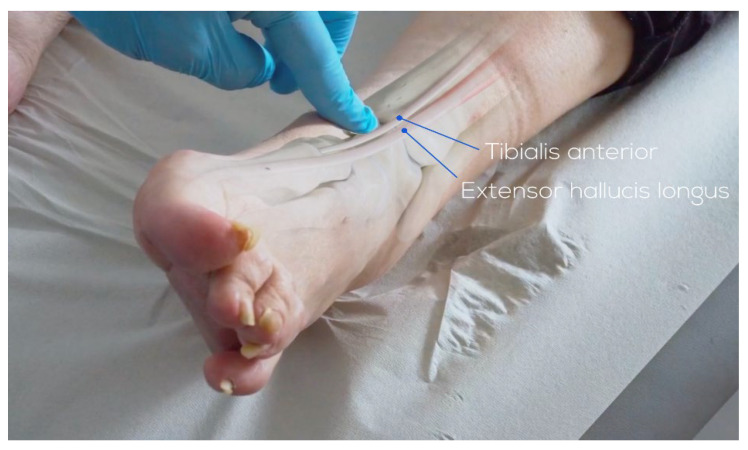
Landmark and skin-incision anterior approach (©Osgenic, Osgenic Oy, Helsinki, Finland, https://osgenic.com/).

**Figure 3:**
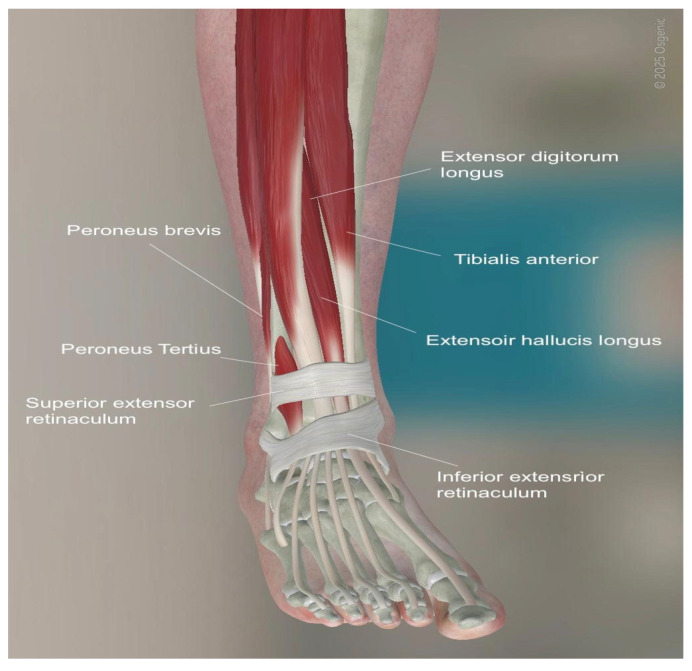
Anatomy of the anterior approach for TAR. (©Osgenic).

Osteophytes are removed from the tibia and the talar neck. Tibial resection is performed, maintaining a 2° to 4° slope on the tibial plafond, and careful technique is used to avoid malleolar fractures, which can occur in up to 10% of cases. A measuring gauge is used to determine the size of the tibial component. After the tibial cut has been performed, approximately 2 mm of bone is removed from the medial side of the talus. The size of the talar component is confirmed to match the previously determined tibial component, with no more than a one-size difference. The posterior capsule is carefully debrided, and any ossifications are removed. After the metallic trial components have been inserted, soft-tissue tension is checked. Finally, the definitive components are inserted and a careful wound closure is performed (Figure 2 and Figure 3).

The range of motion in dorsiflexion and plantar flexion is assessed to ensure no “overstuffing” has occurred, and the periarticular collateral ligaments are adjusted as needed.

### 4.2. Lateral Approach: Indications and Surgical Technique

For patients with an ankle-joint disease due to post-traumatic, rheumatoid, or primary OA, the lateral approach is indicated. In cases of high risk of wound complication, the lateral approach is recommended. The anatomic center of rotation can be identified with ease using this method, and coronal-plane deformities can be addressed without release or significant reconstruction of the deltoid ligament [34].

Usually, the lateral approach is performed under spinal anesthesia and with a pneumatic bandage at thigh level in the absence of specific contraindication. The patient is positioned in a lateral decubitus position on the healthy side. A longitudinal cutaneous incision approximately 15 cm in length is performed laterally at the distal third of the fibula, curving anteriorly below the external malleolar apex. The fibula and the anterior side of the distal tibiofibular syndesmosis are exposed by subperiosteal dissection. After the antero-inferior tibiofibular and interosseus ligaments have been identified and removed, a fibular osteotomy is performed approximately 6–7 cm proximal to the external malleolar apex to provide direct lateral access to the ankle joint. The distal stump of the fibula is therefore temporarily fixed to the heel with a Kirschner wire. The osteotomy is performed obliquely from superolateral to inferomedial to ensure a wider contact area for the subsequent synthesis and to address possible deformities in the coronal plane [35].

**Figure 4:**
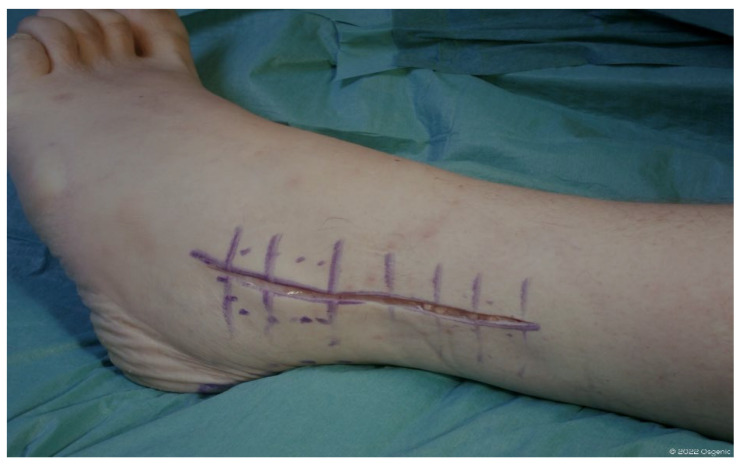
Landmark and skin incision lateral approach (©Osgenic).

**Figure 5:**
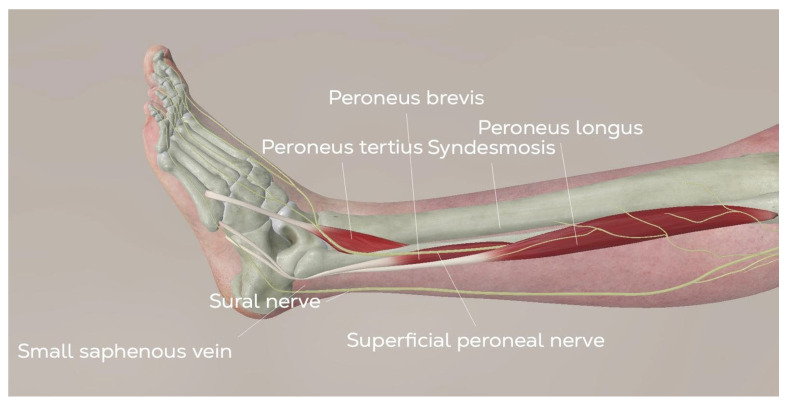
Anatomy of the lateral approach for TAR. (©Osgenic).

The posterior capsule along the posterior tibia and talus is released to bring the ankle to a neutral position at 90°. The patient is then repositioned in a supine position, with the affected limb slightly intra-rotated. The mechanical axis of the ankle is evaluated via X-ray. After the correct positioning of the ankle has been determined, the central axis is identified. The presumed size of the implant and the desired rotation center of the joint are simulated, and bone resections are performed with X-ray confirmation. After stability tests and fluoroscopic checks with temporary components, the definitive components and the most appropriate insert are implanted. Finally, the fibula is reduced and synthesized with interfragmentary screws or a plate and screws (Figure 4 and Figure 5).

## 5. Post-Operative Rehabilitation

Post-operative immobilization and rehabilitation after TAR is crucial and can vary depending on the approach used.

For the anterior approach, to ensure the proper healing of the extensor tendon retinaculum, it is recommended to avoid active dorsal extension in the first 4 weeks after surgery. The foot is confined to a stabilizing walker or cast for 6 to 8 weeks until it is dry and free of any secretions. Weight-bearing is permissible as long as it is safe, except for patients who have undergone more corrective osteotomies.

For the lateral approach, postoperatively, the leg is placed into a cast with no weight-bearing for 4 weeks. Then weight-bearing with a walker-boot is allowed for two weeks.

After immobilization, range-of-motion (ROM) exercises are fundamental to restoring joint functionality and preventing ankle stiffness.

Laser therapy may be used for analgesic and bio-stimulating effects. Active toe flexion and extension can be introduced. Passive movements to restore ROM are useful. Soft-tissue treatment and scar treatment should be included. When sufficient passive ROM has been restored (5–10° in dorsal flexion and 15° degrees in plantar flexion), active exercises should be performed in the open kinetic chain to regain anterior and posterior leg-muscle strength. Gradually, patients can introduce closed-kinetic-chain exercises for plantar flexors and standing on toes. Exercises for proprioception and balance should be introduced at this time. After these movements are restored, walking can be introduced and walking distance can be gradually increased. Activities such as cycling and swimming are admitted in an advanced phase of rehabilitation [36,37].

## 6. Anterior Approach

### 6.1. Functional Outcomes and Survivorship Rate

The anterior approach has a positive impact as judged by improvement in pain and function, as well as by improved gait and increased range of movement [38]. MB and FB implants are usually used for the anterior and lateral approaches, respectively. A comparison of implant type according to the surgical approach was performed by Usuelli et al. [18]. It is important to assess the β angle when treating the malalignment of the ankle. This angle is identified by the longitudinal axis of the tibia and the articular surface of the tibial component in the lateral view; normal values are 85° ± 2°. Determination of this angle is essential to correcting the deformity of the ankle in the sagittal plane. The β angle is less anatomical in the MB group but the analysis of the tibial slope showed that the MB group demonstrated more reproducibility than did the FB group. However, the FB group showed a more anatomic placement of the implant. MB systems, characterized by a polyethylene spacer that allows greater articulation, helped lower shear forces at the bone−implant interface [39]. These designs were more prone to subluxation and malleolar impingement but reduced the risk of loosening [40]. FB implants, which offered more stability and reduced risk of spacer subluxation, underwent substantial refinements to improve their longevity and functional outcomes. They also show more surface damage posteriorly, indicating potential constraints during gait or issues with surgical technique [41,42].

Early outcomes following TAR demonstrate significant improvement in patient-reported outcomes (PROs) across all major implants, including the Scandinavian Total Ankle Replacement (STAR)™, Scandinavian Total Ankle Replacement (Stryker Orthopaedics, Mahwah, NJ, USA), Hintegra ^®^ Total Ankle Prosthesis (Newdeal SA, Lyon, France), Salto-Talaris ^®^ Total Ankle Prosthesis (Tornier SA, Saint Ismier, France), INBONE c Total Ankle System (Wright Medical Technology, Inc., Arlington, TN, USA), and INFINITY ^®^ Total Ankle System (Stryker Orthopaedics, Mahwah, NJ, USA). For the STAR implant, early follow-up studies report consistent improvements in visual analog scale (VAS), short-form 36 (SF-36), and American Orthopedic Foot and Ankle Society (AOFAS) scores, along with high survivorship rates of 94–96% [43]. Similarly, the Hintegra implant shows marked improvements in PROs such as AOFAS, VAS, and SF-12, with survivorship as high as 91–97% [44]. The Salto-Talaris implant also produces excellent early outcomes, including improvements in foot and ankle ability measurement (FAAM) and VAS scores, together with survivorship rates of 98.1–99% [45]. Both the INBONE and the INFINITY systems yield significant improvements in PROs, including AOFAS, short musculoskeletal-function assessment (SMFA), and SF-36, with early survivorship rates ranging from 94–100% [11,46].

Midterm outcomes reveal sustained functional improvements and high patient satisfaction for all TAR implants, though survivorship begins to vary. The STAR implant maintains positive results in PROs such as VAS and ankle OA score (AOS), with survivorship ranging from 78–100% [6,32]. The HINTEGRA is associated with consistent improvements in UCLA activity scores, AOFAS, and VAS, with midterm survivorship reported at 74–81% [33,47,48]. The Salto-Talaris is associated with strong functional outcomes, with survivorship rates of 95.6–100% [49,50]. The INBONE-II outperforms its predecessor, with midterm survivorship of 97–100% [51], while the INFINITY sustains significant improvements in Foot and Ankle Outcome Score (FAOS) and AOS scores, with survivorship rates of 93–100%.

Survivorship over the long term gradually declines, although PROs often remain favorable. The STAR implant shows a measurable decrease in survivorship beyond 10 years, with values ranging from 63.6–87.8%, but patients report continued improvements in AOFAS, VAS, and EuroQol EQ-5D scores. The HINTEGRA implant is associated with survivorship of 82–93.5% at long-term follow-up, with durable gains in AOFAS and VAS scores [52,53]. Long-term data regarding outcomes following the use of the Salto-Talaris implant, INBONE implant, INBONE II implant, and INFINITY implant are limited.

### 6.2. Complications

Usuelli et al. [19] compared 81 patients who underwent TAR with an anterior approach to 69 patients who underwent TAR with a lateral approach. In the anterior group, there was a higher infection rate; in detail, the authors reported three (4%) cases of deep infection and four (5%) of superficial infection, which were treated with vacuum-assisted closure (VAC) and antibiotic therapy. Only one patient underwent revision of the tibial component. Gagné et al. analyzed the reoperation profile of the lateral versus the anterior approach in TAR. The authors showed that even though the anterior surgical approach showed a greater risk of reduced soft-tissue coverage, the risk of deep infection requiring revision surgery was lower than that associated with the lateral approach [12].

Intraoperative fractures, in particular malleolar fractures, are a significant concern in the anterior approach but are rarely a long-term concern if properly recognized and fixed. These fractures often occur during osteotomies of the tibia or talus and can be attributed to biomechanical factors such as poor bone quality, excessive force, or inadequate preparation of the bone. Additionally, most intra-operative fractures are caused by iatrogenic factors; they typically result from insufficient exposure due to the jig or resection guide size, as well as from unintentional use of the saw blade [54]. A recent meta-analysis by Hermus et al. reported an intra-operative fracture incidence of 5.6%, with the medial malleolus being particularly vulnerable during bone resection and implant insertion [55]. Postoperative fractures occur infrequently following TAR, with an incidence rate of 0.03%, and typically result from excessive stress on weakened bone, decreased medial malleolar width, or inadequate implant fixation. They can adversely impact patients’ long-term outcomes, potentially necessitating reoperations, including conversion to ankle fusion in severe cases [55,56].

Aseptic loosening is a significant complication following TAR, with a reported incidence rate of 5% [55]. Aseptic loosening occurs when the bond between the bone and implant fails, often due to poor osseointegration, malalignment, or excessive stress on the tibial and talar components. Factors such as poor bone quality, implant design, inadequate bone preparation, and excessive weight-bearing forces contribute to the development of aseptic loosening [57].

Laceration of posterior ankle structures may occur intraoperatively during tibial or talar resection. Neurovascular injuries are relatively rare complications following TAR, with an incidence of just 0.4% [55]. Intraoperative nerve injury, such as damage to the superficial and deep peroneal nerve and to the posterior tibial nerve, was reported due to the proximity of these critical structures. In some cases, motor and sensory deficits, such as tarsal tunnel syndrome, can occur. These injuries can often be managed with conservative treatment, though severe cases may require nerve repair or even implant revision if the nerve injury significantly impairs function.

In the early postoperative period, wound-healing problems were reported in 2.4–4.0% of cases; this complication can lead to further surgery requiring skin grafts [58,59,60]. The anterior approach provides a direct route to the ankle joint, but the proximity of the incision can place the surgical site at risk for infection due to potential compromise of the anterior tibial vessels. Diabetes mellitus was the only factor that had a significant association with the occurrence of minor wound complications in a review of 12 independent factors that could influence wound healing. Patients with inflammatory connective-tissue diseases (such as rheumatoid arthritis) had an increased risk of developing a major wound complication, with 14-fold increase in the odds of complications necessitating reoperation [61].

Anterior ankle and gutter impingement are uncommon complications following TAR, occurring in 0.06% of cases [55]. They typically result from soft-tissue or bony impingement in the medial or lateral gutters, with factors such as malpositioned components, subsidence, hypertrophic bone formation, or improper alignment contributing to their development [62]. Heterotopic ossification (HO), the abnormal formation of bone in soft tissues, is a recognized cause of impingement and has been noted in approximately 44.6% of cases [63]. HO can exacerbate impingement by contributing to gutter pain, stiffness, and functional limitations, occasionally necessitating surgical excision to relieve symptoms and improve joint mobility. While impingement may require revision surgery to address malalignment or hypertrophic bone, preventive strategies such as gutter widening and precise component positioning during the initial procedure can reduce its occurrence [55].

Other complications reported in the literature include subsidence of the talar component, cyst formation, severe instability, and painful arthrofibrosis [64].

## 7. Lateral Approach

### 7.1. Functional Outcomes and Survivorship Rate

If wound complications occur, there is no direct exposure to the implant, which is one of the main advantages of the lateral approach. Despite this, the lateral plate, situated on the fibula, would be visible. The literature advises that a long oblique fibular osteotomy be performed and secured with free 3.5 mm cortex screws, thus preventing plate exposure in case of wound-healing issues.

Considering the type of implant, the Zimmer Trabecular Metal Total Ankle Replacement (TM TAR) ™ (Zimmer, Warsaw, IN, USA) has shown promising clinical outcomes in both short- and mid-term follow-ups, yielding significant improvements in pain relief, range of motion, and patient satisfaction, with high early survivorship. Barg et al. reported a 93% implant survival rate at 2 years, with substantial reductions in pain scores and increased ankle range of motion [65]. Similarly, a multicenter study by D’Ambrosi et al. showed significant early functional improvements with regard to AOFAS, EQ-5D, AOS pain, and AOS difficulty scores at 6 weeks, 6 months, 1 year, 2 years, and 3 years postoperatively, together with a 97.4% Kaplan-Meier survival estimate at 3 years [66].

Midterm outcomes following TM TAR indicate sustained improvements in function and pain relief, with generally favorable survivorship, though complications such as reoperations and osteolysis are reported. Tiusanen et al. assessed post-operative outcomes following TM TAR in 104 patients at a mean follow-up of 43.6 months, where 89% of patients demonstrated improved function, with 66% expressing high satisfaction post-surgery. Furthermore, 50% of patients reported no pain and the mean Kofoed score improved significantly, from 37.6 at baseline to 74.8 at final follow-up [67]. Fletcher et al. evaluated 83 ankles (81 patients) undergoing TM TAR at a mean follow-up of 6.3 years [34]. Overall, there were significant improvements in ankle range of motion, alignment, and low pain levels, with postoperative SF-12 Physical Component Summary (PCS) scores averaging 40.4 and postoperative VAS scores averaging 2.3.

Fa-Binefa et al. conducted a systematic review of 919 individuals who underwent TM TAR at a mean age of 62 years, where 49% were women and the main etiology of ankle OA was posttraumatic (55%). The study found that patients who underwent TM TAR showed significant clinical improvements at a mean follow-up of 3 years, with a mean VAS score of 4.2 and a 53.7 point improvement on the AOFAS scale. The mean weighted survivorship of TM TAR components at the final follow-up was 97% [13].

### 7.2. Complications

An important issue addressed by several studies is the infection rate and the need for surgical re-intervention [68]. A study published by Usuelli et al. [19] compared the two approaches to analyze the rate of post-operative infections. In detail, the study compared the rates of superficial and deep infections in the two types of surgical approach over a 12-month follow-up period; the authors showed that, despite the average surgical time of 179 min in the lateral-approach group, there was a lower rate of infection: only one (1.4%) case of deep infection, which necessitated the removal of the prosthesis and implantation of an antibiotic spacer, and two (2.9%) cases of superficial infection, which were treated with antibiotics and VAC therapy. In conclusion, the rates of superficial and deep periprosthetic infections were not significantly different between the groups despite a significantly longer surgical time reported for the lateral approach group.

In contrast, a further study by Gagné et al. [12] showed that the lateral approach had a higher rate of postoperative complications requiring further surgical treatment. In detail, 22 out of 48 patients had complications that required revision surgery; eight of these patients underwent another revision surgery, and three patients underwent further additional surgery. Common reoperations were related to the lateral fibula hardware or HO debridement.

One of the proposed advantages of the lateral transfibular approach is the reduced risk of iatrogenic medial malleolar fractures; in fact, the transfibular approach, by accessing the joint through a lateral incision, avoids direct manipulation of the medial malleolus, reducing the risks of such fractures [55].

Compared to the anterior approach, the lateral approach typically avoids neurological injury to the deep peroneal nerve and anterior tibial vessels. Patients may report plantar pain and numbness localized to the distributions of the medial and lateral plantar nerves, resulting in substantial discomfort and functional impairment and potentially impacting the patient’s ability to walk or perform daily activities. Any disruption or compression of the tibial nerve within this tunnel can lead to neuropathic symptoms, particularly when mechanical factors such as surgical retractors or altered positioning during correction of deformities place undue stress on the nerve. In cases of significant varus-deformity correction, the surgical exposure often requires retraction of the surrounding soft tissues and nerves, which may increase the risk of injury to the tibial nerve. Clugston et al. identified three cases (3.5%) of injury to the tibial nerve in their cohort. Intermittent relaxation of retractors during the preparation of bony cuts may reduce the incidence of prolonged tibial-nerve compression, thus reducing the risk of iatrogenic injury [68]. A further issue with the lateral approach is potential injury to the first perforator of the peroneal artery.

Other complications reported include delayed union and non-union of lateral osteotomy of the fibula, which can lead to functional problems and often to further surgery [69]. Also, if a complete revision of the ankle is necessary, the fibular osteotomy will need to be rerun and fixed again.

Symptomatic fibular nonunion, occurring in 2% of cases, is a significant complication of the fibular osteotomy associated with the transfibular approach. Factors such as inadequate fixation, the stability of the osteotomy site, and the patient’s bone health can contribute to the risk of fibular nonunion. Although fibular nonunion is relatively rare, its impact can be profound, requiring reoperation or leading to dissatisfaction in patients, particularly if it is managed nonoperatively [13].

## 8. Return to Sport

Return to sport (RTS) after TAR is still a controversial topic in the literature, as there are no guidelines for surgeons. While TAR primarily aims to restore pain-free mobility and improve quality of life, many patients desire to maintain or resume athletic activity postoperatively. Historically, sports participation was limited following TAR due to concerns about implant durability and the potential impact of high-impact activities on prosthesis longevity. However, advances in implant design, surgical technique, and postoperative protocols have contributed to improved outcomes, prompting growing interest in understanding how these factors influence RTS rates and activity levels. Notably, the type of sport, patient characteristics, and preoperative activity levels play crucial roles in determining postoperative sports participation, as do the expectations set during preoperative counseling. To date, RTS has been assessed in studies of implants using the anterior approach, with no RTS data published following the use of the lateral transfibular approach. Valderrabano et al. analyzed RTS following TAR and reported that 76% of participants maintained their sports activities postoperatively, while 44% of previously inactive patients regained the capacity for sports participation [70]. Similarly, Naal et al. conducted a prospective analysis of 124 patients and observed a 92.1% RTS rate, with 61.3% of participants reporting improved sports ability postoperatively [71]. Macaulay et al. [72] proposed a survey of foot and ankle specialists on physical activity and sports restrictions based on their experience. Twenty-two of the 50 sports and activities proposed, such as dancing, bowling, swimming, and low-impact aerobics, were classified as “allow everyone”; 10 sports, such as hiking, ice skating, and mountain biking, were classified as “only allow patients with significant prior experience”; and the remaining 18 sports, including basketball, jogging, soccer, and volleyball, were classified as “do not allow”. For younger patients (<50 years old), some surgeons were more restrictive after surgery, whereas 39% did not change their restrictions. For older patients (>70 years old), 46% of the surgeons were less restrictive. Factors such as BMI > 30 km/m^2^ and poor quality of bone made the surgeons more restrictive postoperatively. Surgeons were comfortable with low-impact sports, especially for older, non-obese patients with good bone quality. Sports with boot immobilization represent a grey area, and the decision of what to allow depends on the prior experience of the patient.

The return to sport after TAR versus arthrodesis was compared by Johns et al. [73], with a total of 1270 ankle procedures analyzed. About 64% of patients were active in sports after surgery, compared to 55% before surgery. There were no differences between TAR and arthrodesis in return to low-impact sports such as cycling, swimming, and dancing, but patients who underwent TAR experienced earlier improvements in physical-activity levels in comparison to patients who underwent arthrodesis. Soccer, running, tennis, and basketball were the most common sports that patients were unable to participate in after surgery, as they were not well-tolerated [74,75]. An individual approach for TAR should consider age, preoperative activity level, and previous experience in that sport when making recommendations for the return to moderate- or high-impact sports postoperatively. Vertullo and Nunley et al. [76] investigated the type of sports recommended by the surgeons after an arthrodesis procedure, finding that only golf and skiing were permitted.

A systematic review conducted by Arceri et al. [77] focused on the return to sport after TAR. In four studies [70,71,78,79], 49% of patients were active in sports before surgery, but after surgery, this percentage rose to 62%. None of the patients performed sports at a competitive level either before or after surgery, but all of them expressed satisfaction after TAR and returned to their pre-operative activity levels. This review also evaluated the type of sports activities; cycling, hiking, swimming, and gymnastics were the most frequently reported, but, three studies reported higher participation in jogging, downhill skiing, tennis, and skiing after TAR. Finally, 7 of the 11 included studies reported that the type of implant used was the MB, but it seemed that the type of implant did not correlate with better scores on functional outcomes. The current evidence suggests that TAR facilitates a meaningful return to sport for selected patients, albeit predominantly in low-impact activities. While younger, healthier patients with non-inflammatory arthritis achieve the most favorable outcomes, no definitive conclusions can be drawn regarding long-term implant durability and the risks associated with high-demand activities. To date, there has been a lack of data published regarding RTS in patients who underwent TAR via a lateral transfibular approach. The variability in reported RTS rates may be attributed to differences in study populations, methodologies, and follow-up durations, emphasizing the need for standardized outcome measures in future research. Until more robust evidence emerges, clinicians should counsel patients on realistic expectations, emphasizing low-demand activities and individualizing postoperative recommendations to optimize outcomes.

## 9. Limitation of the Study

Several limitations should be considered when interpreting the results of this study. First, our search was limited to studies published in English, which could potentially contribute to publication bias. In addition, four major literature databases were used for the search, so the possibility of finding additional articles using other databases cannot be excluded. Second, the potential for bias introduced by different surgical approaches led us to consider studies different types of implants. Poor implant design, loosening, and instability were the main causes of failure in first-generation cemented TAR, constrained and unconstrained [80]. The search for a successful TAR, therefore, continued, leading to the development of second- and third-generation implants with numerous modifications, including a semi-constrained cementless design and the FB and MB designs, which yield better results than did the first-generation designs [81].

Clinicians should not overlook the possibility of differences in patient characteristics in their search for the best surgical approach for TAR. It is important to select the right patients to optimize outcomes and to select a particular treatment and approach. Moreover, few studies available in the literature evaluated bone quality, quality of life, and mental status [82]. These factors could influence the decision with regard to treatment and approach and have already been associated with impaired functional recovery after various orthopedic procedures [83,84].

## 10. Conclusions

The management of end-stage ankle OA still represents a challenge for surgeons, and TAR is the most cutting-edge surgical technique. The anterior approach with the MB implant restores the normal tibial slope but has a high risk of wound-healing complications and medial malleolar fractures. The lateral approach with the FB implant allows an anatomic placement of the implant but is associated with fibular complications and a high risk of revision surgery. Return to sport is feasible in low-impact sports such as cycling, swimming, and dancing, especially for older, non-obese patients with good bone quality. New studies evaluating patient selection, surgical approaches, and the types of sports are necessary to optimize outcomes after TAR.

## Figures and Tables

**Table 1:** Quality assessment of included studies according to the Modified Newcastle−Ottawa scale.

Study Author (Year)	Criteria								Total	Quality
	1	2	3	4	5	6	7	8		
Gagné et al. (2020) [12]	1	0	1	1	2	1	1	1	8	High
Usuelli et al. (2016) [17]	1	0	1	1	2	1	1	1	8	High
Usuelli et al. (2016) [18]	1	0	1	1	2	1	1	1	8	High
Usuelli et al. (2017) [19]	1	0	1	1	2	1	1	1	8	High
Usuelli et al. (2020) [20]	1	0	1	1	2	1	1	1	8	High

Based on the total score, quality was classified as “low” (0–3), “moderate” (4–6), or “high” (7–9). The criterion numbers (in bold) are as follows: 1, representativeness of the exposed cohort; 2, selection of the nonexposed cohort; 3, ascertainment of exposure; 4, demonstration that outcome of interest was not present at start of study; 5, comparability of cohorts on the basis of the design or analysis; 6, assessment of outcome; 7, whether follow-up was long enough for outcomes to occur; 8, adequacy of follow-up of cohorts. Each study was awarded a maximum of one or two points for each numbered item within categories, based on the Modified Newcastle-Ottawa scale rules.

## Data Availability

Not applicable.
